# Mechanistic study of the effect of flexible fixation and load‐bearing stress environment on fracture healing and shaping

**DOI:** 10.1002/ame2.12448

**Published:** 2024-07-08

**Authors:** Xingfu Li, Zhenhan Deng, Wei Lu

**Affiliations:** ^1^ Department of Sports Medicine Shenzhen Second People's Hospital (The First Affiliated Hospital of Shenzhen University) Shenzhen Guangdong China; ^2^ Guangdong Key Laboratory for Biomedical Measurements and Ultrasound Imaging, National‐Regional Key Technology Engineering Laboratory for Medical Ultrasound, School of Biomedical Engineering Shenzhen University Medical School Shenzhen Guangdong China; ^3^ Department of Orthopedics Surgery The First Affiliated Hospital of Wenzhou Medical University Wenzhou Zhejiang China

**Keywords:** CXCL12, flexible fixation, sonic hedgehog, stress environment

## Abstract

**Background:**

The biomechanical environment created by suture‐button fixation Latarjet is conducive to the healing and shaping of the transplanted coracoid, but its mechanism remains unclear. The latest research has found that the absence of stem cell chemokine (CXCL12) impeded bone regeneration in Sonic Hedgehog (SHH)‐deficient animals. However, whether the biomechanical environment affects SHH and CXCL12 function has not been studied.

**Methods:**

Rat fracture models were constructed to simulate stress environments under non‐load‐bearing and load‐bearing conditions. The fracture healing and shaping, as well as the expression levels of SHH and CXCL12, were assessed through gross viewing, micro‐computed tomography (micro‐CT), and histochemical staining.

**Results:**

Under flexible fixation, the relative bone volume (BV/TV) of rats exposed to the load‐bearing stress environment was significantly higher than that of rats under a non‐load‐bearing stress environment (*p* ≤ 0.05). Adverse bone shaping was not observed in rats subjected to flexible fixation. The levels of SHH and CXCL12 in load‐bearing rats exhibited significant elevation (*p* ≤ 0.05). Under a load‐bearing stress environment, no significant difference was observed in the BV/TV between the flexible fixation group and the rigid fixation group (*p* ≥ 0.05), but there was excessive hyperplasia of the fracture callus in the rigid fixation group. The levels of SHH and CXCL12 in rats subjected to rigid fixation were significantly elevated (*p* ≤ 0.05).

**Conclusions:**

Flexible fixation and load‐bearing stress environment may contribute to bone healing and shaping by influencing the levels of SHH and CXCL12, suggested that this mechanism may be relevant to the healing and shaping of the transplanted coracoid after suture‐button fixation Latarjet.

## Graphical abstract

The biomechanical environment created by modified suture‐button fixation (MSBF) arthroscopic Latarjet is conducive to the healing and shaping of the transplanted coracoid, but its mechanism remains unclear. Recent research has shown that Sonic Hedgehog (SHH) is the fundamental driving factor in bone healing, with the absence of stem cell chemokine (CXCL12) proving detrimental to bone regeneration in animals lacking SHH. However, whether the biomechanical environment affects SHH and CXCL12 function has not yet been studied. We constructed fracture models and simulated stress environments under non‐load‐bearing and load‐bearing conditions. The fracture healing and shaping, the expression level of SHH and CXCL12, and the recruitment of stem cells were evaluated by gross viewing, micro‐computed tomography (micro‐CT), and histochemical staining. Under flexible fixation, the relative bone volume (BV/TV) of rats in a load‐bearing stress environment was significantly higher than that of rats in a non‐load‐bearing stress environment (*p* ≤ 0.05). Adverse bone shaping was not observed in rats with flexible fixation. The SHH and stem cell recruitment levels of load‐bearing rats were significantly higher (*p* ≤ 0.05). Under a load‐bearing stress environment, no significant difference was observed in the relative bone volume (BV/TV) between the flexible fixation group and the rigid fixation group (*p* ≥ 0.05), but there was excessive hyperplasia of the fracture callus in the rigid fixation group. The SHH and stem cell recruitment levels of rats subjected to rigid fixation methods were significantly higher (*p* ≤ 0.05). In conclusion, flexible fixation and a load‐bearing stress environment may contribute to bone healing and shaping by influencing the levels of SHH and stem cell recruitment, suggesting that this mechanism may be involved in the healing and shaping of transplanted coracoid after MSBF arthroscopic Latarjet.

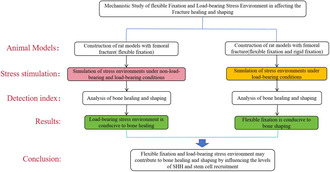

## 1 INTRODUCTION

The Latarjet procedure has remained the gold standard in managing recurrent anterior shoulder instability (RASI) for over seven decades.^1^ The rigid fixation method of traditional Latarjet uses screws for coracoid bone graft fixation and has demonstrated efficacy in RASI. However, this approach is associated with several drawbacks and complications, including a stress shielding effect, additional tissue damage, complicated operations, postoperative bone nonunion, excessive bone resorption, graft malpositioning, as well as potential trauma or graft fracture.^2^, ^3^, ^4^

In 2012, Boileau et al. improved the technique by fixing the coracoid bone graft with suture‐buttons, a form of flexible fixation, to avoid screw related complications, which achieved better clinical and imaging results.^5^ This method of coracoid bone graft has been demonstrated in our previous studies to exhibit expansion and remodeling during the healing process, with minimal bone resorption and satisfactory clinical outcomes.^6^, ^7^, ^8^, ^9^

However, the mechanism underlying the promotion of bone healing through flexible fixation remains elusive, and a well‐established animal model for Latarjet surgery is yet to be reported in the literature. Several studies have demonstrated that mechanical stress can enhance the proliferation, differentiation, and migration of mesenchymal stem cells (MSCs) while expediting fracture healing. These findings imply a potential association between biomechanical environment and stem cell recruitment.^10^, ^11^ Knocking out SHH, a fundamental regulator of bone healing, significantly impedes osteogenesis and reduces CXCL12 expression, thereby impairing stem cell recruitment and ultimately affecting the process of bone healing. These findings suggest a potential synergistic interplay between CXCL12 and SHH.^12^, ^13^ In this study, we employed both rigid and flexible fixation following femur fracture to investigate the potential impact of flexible fixation on bone healing and shaping by comparing the bone volume/total volume (BV/TV) in rat models of femur fracture under different mechanical loading conditions, and we explored the mechanism by comparing the SHH and CXCL12 levels of femur fracture model in rats under different fixation strengths and stress environments.

## 2 MATERIALS AND METHODS

All the animal experiments were approved by the Shenzhen Second People's Hospital, China Technology Industry Holdings (Shenzhen) Co., Ltd (IRB no: 202200112) and performed in accordance with the national guidelines for animal welfare.

### 2.1 Femur fracture model

Sprague Dawley rats were placed in the lateral decubitus position after induction using isoflurane. The bilateral femurs were operated in this animal experimental model. The operation area was disinfected using a compound disinfection solution of povidone iodine (0.1%) at least three times after skin preparation. The femurs were exposed via the femur lateral approach. The femurs were cut in the middle by an electric cranial drill to construct the fracture model and then the fracture sites were fixed with Kirschner wire or plate screws. Finally, the operative incision was sutured layer by layer. After the operation, the rats were routinely bred for 1 week before being treated with different stress environments. The surgical site was disinfected with 0.1% povidone iodine daily in the following 2 weeks.^14^ The rats were divided randomly into five groups (*n* = 6/group): a normal control group (NC), a sham control group that underwent lateral femoral incision exclusively (SC), a flexible fixation group with load‐bearing stress environment (FFL), a flexible fixation group with non‐load‐bearing stress environment (FFNL), and a rigid fixation group with load‐bearing stress environment (RFL).

### 2.2 Hindlimb unloading

On the eighth day following the surgery, rats were either subjected to hindlimb unloading through tail suspension or kept unsuspended (*n* = 6 per treatment group) for 60 days. Each suspended and control animal was single‐housed in a GMP environment. Tail suspension experiments were performed with standard housing conditions as above.^15^ After sacrifice, the femurs were dissected out and processed for micro‐CT and histomorphometry, as described in the following sections.

### 2.3 Gross viewing and micro‐CT

After 60 days of different stress stimulation, all rats were sacrificed by carbon dioxide euthanasia. The femurs were isolated and fixed in 4% paraformaldehyde solution for 72 h. We obtained a gross view of femur healing and shaping from microphotography.^16^ The micro‐CT imaging was conducted using an animal scanner at a medium resolution, with a voxel size of 25 μm, voltage set at 70 kVp, and a current of 200 μA. The mineral content was quantified within the central 2.50 mm (100 scan slices).^17^ The threshold for bone callus was established at 50% of the average mineral density of native cortical bone, based on previous studies and visual analysis of multiple CT scan slices, in order to ensure that this criterion effectively captured newly formed bone callus.

### 2.4 Histological evaluation of bone healing and shaping

The femur specimens were fixed in 4% paraformaldehyde for at least 72 h and subsequently decalcified in 20% EDTA buffer (pH = 7.4) for 14 days. Subsequently, tissues were embedded in paraffin. Serial sections (5 μm) in the sagittal view were stained with hematoxylin & eosin (HE) staining and Masson's trichrome staining to assess the general morphology at the fracture site.^18^ Masson staining of bone tissue was semiquantitatively analyzed using Image‐Pro Plus 6.0 software (Media Cybernetics, Rockville, MD, USA). For immunohistochemistry, the paraffin‐embedded sections were processed and stained with rabbit anti‐SHH, or anti‐CXCL12 antibodies (Abcam, Waltham, MA, USA), then with the Diaminobenzidine (DAB) Chromogen Kit (Servicebio, Wuhan, China).^19^ Mean optical density (MOD) values were acquired using Image‐Pro Plus 6.0 software by dividing the summation of the optical density of each pixel in the area by the area sizes.

### 2.5 Statistical analysis

All data are presented as mean ± standard deviation (SD). Statistical analysis was performed using GraphPad Prism software, version 8.02 (GraphPad Software, San Diego, CA, USA). Statistical significance comparing two groups with parametric data was assessed by two‐tailed *t* test. Statistical analysis comparing multiple groups with parametric data was performed by one‐ or two‐way ANOVA analysis with Tukey's post hoc test. A value of *p* ≤ 0.05 was considered statistically significant.

## 3 RESULTS

### 3.1 Gross views of femur healing and shaping

**FIGURE 1:**
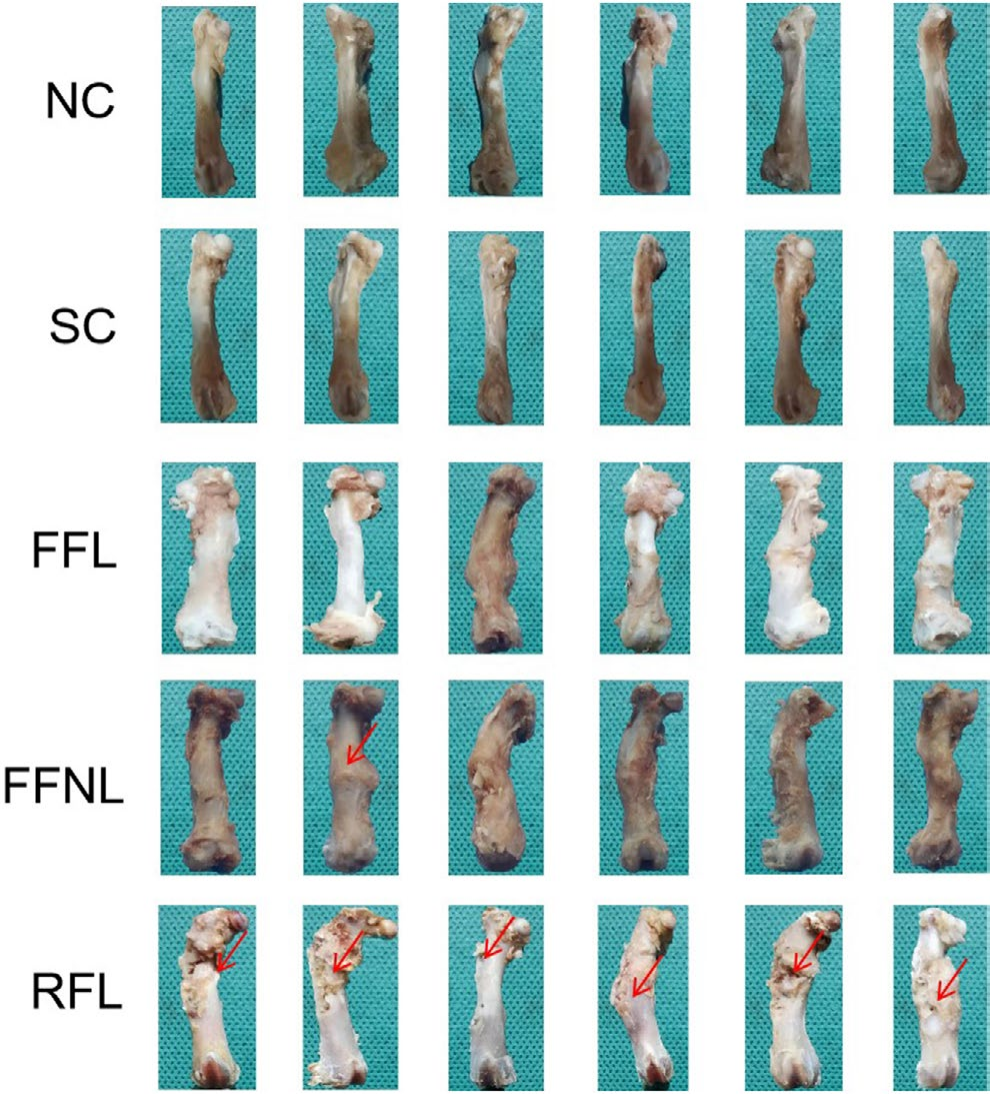
Gross views of femurs healing and shaping (*n* = 6/group). Red arrows indicate the excessive hyperplasia of bone callus. FFL, flexible fixation group with load‐bearing stress environment; FFNL, flexible fixation group with non‐load‐bearing stress environment; NC, normal control group; RFL, rigid fixation group with load‐bearing stress environment; SC, sham control group.

The pictures taken by microphotography revealed that bone healing and shaping were satisfactory under flexible fixation, but excessive hyperplasia of the fracture callus was observed in the RFL group (Figure 1). Thus, the results suggested that flexible fixation might contribute to bone shaping.

### 3.2 Micro‐CT analysis of femurs

**FIGURE 2:**
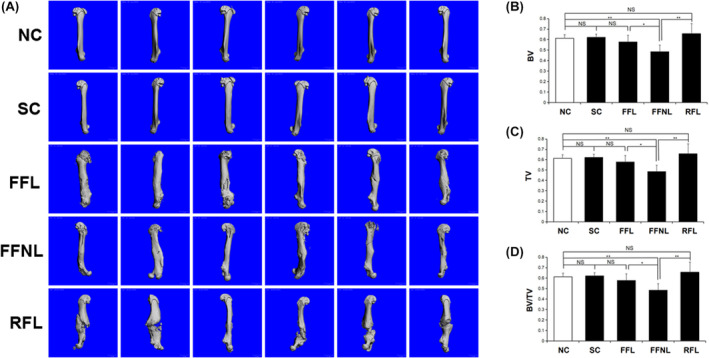
Micro‐CT assessment of femurs (*n* = 6/group). (A) 3D image of femurs. (B) Micro‐CT analysis of the BV. (C) Micro‐CT analysis of the TV. (D) Micro‐CT analysis of the BV/TV. FFL, flexible fixation group with load‐bearing stress environment; FFNL, flexible fixation group with non‐load‐bearing stress environment; NC, normal control group; NS, no significance; RFL, rigid fixation group with load‐bearing stress environment; SC, sham control group; *p < 0.05; **p < 0.01.

The three‐dimensional (3D) imaging of femurs indicated that the RFL group exhibited excessive hyperplasia in comparison to the other groups (Figure 2A). The micro‐CT results revealed a significantly higher bone tissue volume (BV) in the RFL group, compared to the NC group (*p* ≤ 0.05, Table 1; Figure 2B). However, the tissue volume (TV) of the FFL group surpassed that of the NC group (*p* ≤ 0.05, Table 1; Figure 2C). The TV, BV, and BV/TV of the RFL group were similar with that of the FFL group (*p* ≤ 0.05, Table 1; Figure 2B–D). The bone mineral density (BMD) of the FFNL group was found to be significantly lower than the other groups (*p* ≤ 0.05, Table 1; Figure 2D).

**TABLE 1:** Micro‐CT analysis of femurs.

Groups	BV (AV ± SD)	TV (AV ± SD)	BV/TV (AV ± SD)
NC	32.0243 ± 3.5605	52.5677 ± 7.4679	0.6120 ± 0.0352
SC	34.7164 ± 4.7118	56.0249 ± 8.3546	0.6215 ± 0.0311
FFL	37.5463 ± 7.0221	65.5567 ± 11.9910	0.5750 ± 0.0666
FFNL	29.5389 ± 7.3479	61.1562 ± 13.4343	0.4846 ± 0.0632
RFL	39.7380 ± 7.4824	61.3435 ± 13.3708	0.6573 ± 0.0943

*Note*: The relative bone volume (BV/TV) represents the ratio of bone tissue volume (BV) to tissue volume (TV), which directly reflects the change of bone mass.

Abbreviations: AV, average value; SD, standard deviation.

### 3.3 Histological assessment of femurs

**FIGURE 3:**
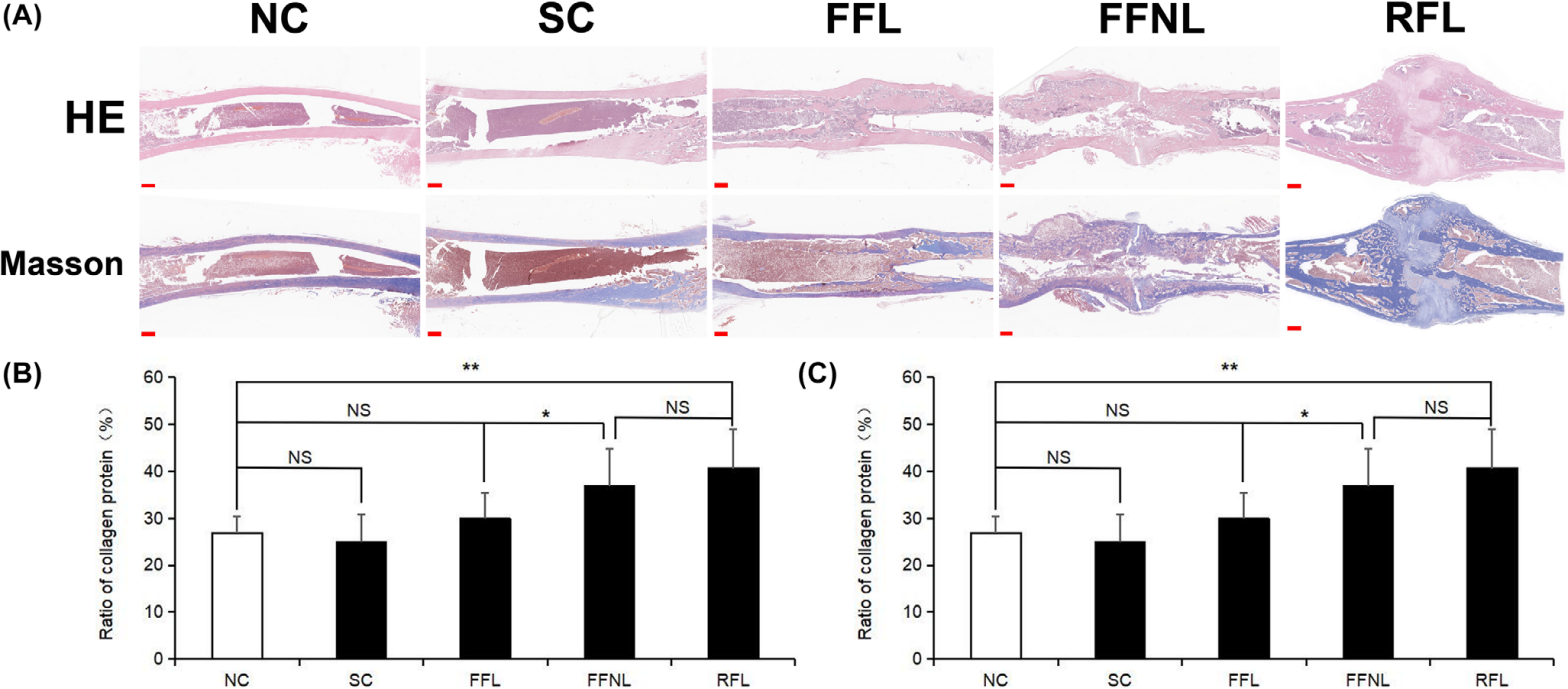
Histological analysis of femurs (*n* = 6/group). (A) HE and Masson staining results of femurs, Scale bar = 1000 μm, red arrows indicate the location of bone callus formation. (B) Image J analysis of HE staining. (C) Image J analysis of Masson staining. FFL, flexible fixation group with load‐bearing stress environment; FFNL, flexible fixation group with non‐load‐bearing stress environment; NC, normal control group; NS, no significance; RFL, rigid fixation group with load‐bearing stress environment; SC, sham control group; *p < 0.05; **p < 0.01, ***p < 0.001.

The femur structure in the FFL group was similar to NC group (Figure 3A). In contrast, the RFL group exhibited excessive callus hyperplasia (Figure 3A). The HE staining revealed a significantly greater diameter of the fracture site in the RFL group compared to the other groups (*p* ≤ 0.001, Figure 3B). Additionally, a slightly larger diameter of the fracture site was observed in the FFL and FFNL group compared to the NC group (*p* ≤ 0.05, Figure 3B). The Masson staining revealed a significantly higher proportion of collagen in both the FFNL group and the RFL group compared to the NC group (*p* ≤ 0.01, Figure 3C). However, there was no significant difference in collagen proportion between the FFL group and the NC group (*p* ≥ 0.05, Figure 3C).

### 3.4 Immunohistochemical assessment of femurs

**FIGURE 4:**
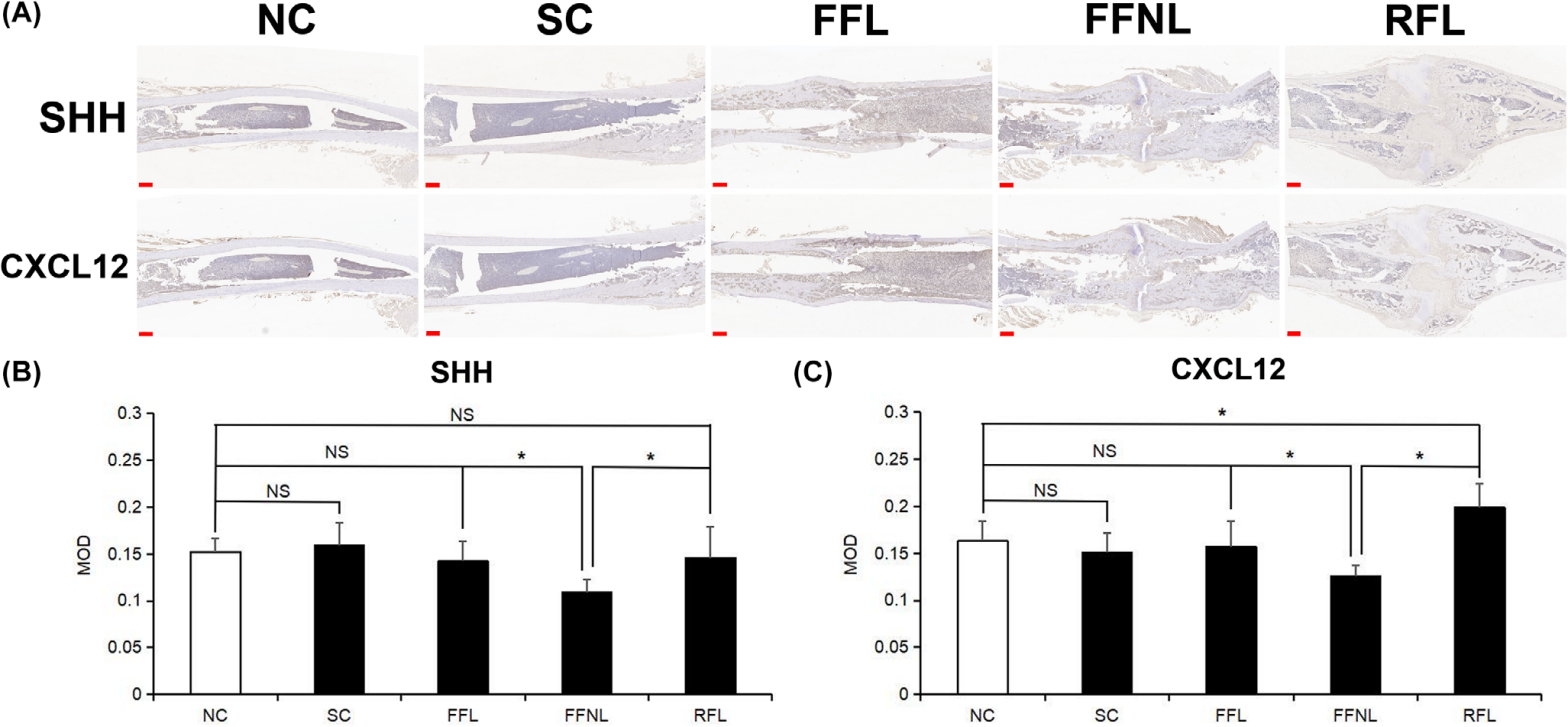
Immunohistochemical analysis of femurs (*n* = 6/group). (A) SHH and CXCL12 staining results of femurs, Scale bar = 1000 μm. (B) Image J analysis of SHH staining. (C) Image J analysis of CXCL12 staining. FFL, flexible fixation group with load‐bearing stress environment; FFNL, flexible fixation group with non‐load‐bearing stress environment; NC, normal control group; NS, no significance; RFL, rigid fixation group with load‐bearing stress environment; SC, sham control group; *p < 0.05.

As shown in Figure 4, the non‐load‐bearing stress environment resulted in decreased MOD of SHH and CXCL12 staining in bone callus tissue compared to the other groups (*p* < 0.05, Figure 4). These findings are opposite to the results in the RFL or FFL group (*p* < 0.05). The MOD levels of CXCL12 and SHH staining in the FFL group did not exhibit significant differences compared to those in the NC group (*p* > 0.05). However, the MOD of CXCL12 staining was found to be higher in the RFL group compared to the other groups, which corresponded to the excessive hyperplasia of the femur callus (*p* < 0.05, Figure 4A,C).

## 4 DISCUSSION

In this study, we have discovered that flexible fixation in bone surgery and a load‐bearing stress environment can contribute to the maintenance of normal expression levels of CXCL12 and SHH, which are crucial for recruiting stem cells and maintaining bone metabolic balance, thereby facilitating bone healing and shaping.

As described by Wolff's law, pressure can stimulate bone growth.^20^ Bone can be reshaped by changes in size, morphology and structure to adapt to the requirements of force. This effect has been proved over time and applied by other scholars, and at present, scholars have reached a consensus that bone growth can be affected by mechanical stimulation to change its structure.^21^, ^22^ Thus, it is now uncontroversial that stress stimulation is the determinant of bone morphology, while exercise and maintenance of muscle dynamics are the key factors to stimulate bone formation.^23^ Some researchers believe that the callus formed at the fracture site is a specific functional entity, which produces physiological responses to the stress environment and cell active factors.^24^ According to Wolf's law, the bone callus can respond to mechanical stress. Thus, if a suitable mechanical environment is created in surgery, then most fractures will heal and shape smoothly.^25^ The mechanical environment created by suture‐button fixation Latarjet, with no stress shielding effect, may therefore be conducive to the healing and shaping of the transplanted coracoid as described by Wolff's law. This suggests an advantage of suture‐button fixation Latarjet over screw fixation Latarjet in coping with bone healing and shaping. In this study, a gross view of femurs indicated that employing flexible fixation may contribute to bone shaping. Micro‐CT analysis of femurs revealed normal formation of callus tissue in the flexible fixation group, and a higher level of mineralization in the load‐bearing stress environment groups compared to those subjected to no‐load‐bearing stress. HE and Masson staining confirmed the radiological findings of normal callus formation in the flexible fixation group. Image J analysis of HE staining show that the femur structure in the flexible fixation group with load‐bearing stress environment was similar to the normal control group. In contrast, the rigid fixation group exhibited excessive callus hyperplasia. Image J analysis of Masson staining revealed that the bone callus in the flexible fixation group with load‐bearing stress environment exhibited a higher degree of maturation compared to both the non‐load‐bearing stress and rigid fixation groups, approaching levels observed in the normal control group. The results of our study indicate that the flexible fixation and stress environment provided by suture‐button fixation Latarjet is conducive to fracture healing and shaping, which provides theoretical support for the clinical promotion of suture‐button fixation Latarjet.

Many studies have demonstrated that stem cells play an important role in the process of fracture healing and remodeling.^26^ Stem cells not only differentiate into osteoblasts, but can also regulate the biological behavior of osteoclasts in both directions.^27^, ^28^ Therefore, the recruitment of stem cells at the fracture site is essential for bone healing and shaping. In recent years, it has been found that mononuclear macrophages infiltrating the fracture site can secrete CXCL12, which guides stem cells with the CXCR4 receptor to migrate to the fracture site and promote bone healing.^29^, ^30^ Whether the biomechanical environment created by suture‐button fixation Latarjet can affect stem cell recruitment by altering CXCL12 level is unknown. Now, our study has found that flexible fixation and a load‐bearing stress environment can effectively maintain normal expression levels of CXCL12 in bone callus tissue, which means that the analogous biomechanical environment constructed by suture‐button fixation Latarjet may be conducive to the aggregation of stem cells. Our results suggest that suture‐button fixation Latarjet may regulate the expression of CXCL12 by constructing a suitable biomechanical environment to promote the healing and shaping of the transplanted coracoid, which means that the biomechanical environment, cytochemokines and recruitment of stem cells may synergistically contribute to the process of bone healing and shaping.

Sonic Hedgehog (SHH) has been found to be an early and fundamental driver of bone healing, and knockdown of SHH significantly inhibits osteogenesis.^31^ In addition, animals lacking SHH signaling have been found to lack cells expressing CXCL12, suggesting that loss of SHH signaling may raise a barrier in CXCL12‐mediated stem cell recruitment, thereby affecting bone healing and shaping.^13^ Thus, there is a link between SHH, which promotes osteogenesis, and CXCL12, which promotes stem cell recruitment, and both of these cytokines are directed to regulate bone healing and shaping. However, as with CXCL12, whether SHH is affected by the biomechanical environment constructed by suture‐button fixation Latarjet is still unclear. Although it is uncertain whether the biomechanical environment affects coracoid healing and shaping by regulating SHH levels after Latarjet, it is widely accepted that mechanical stress stimulation can promote protein synthesis.^32^ Therefore, it is reasonable to speculate that the biomechanical environment created by suture‐button fixation Latarjet may regulate coracoid healing and shaping by affecting the secretion of SHH. Our study has now revealed that flexible fixation and a load‐bearing stress environment contribute to maintaining normal expression levels of SHH at the fracture site, indicating that flexible fixation and stress stimulation is beneficial for bone formation. The results suggest that suture‐button fixation Latarjet may regulate the expression of SHH by constructing a suitable biomechanical environment to promote the healing and shaping of the transplanted coracoid, as suggested above.

Unexpectedly, it was found in our study that although rigid fixation ensured calcium deposition in the fracture site under the stimulation of loading stress, it resulted in excessive callus formation, which was not conducive to bone shaping, while flexible fixation not only maintained calcium deposition but also avoided callus hyperplasia under the same stress environment. These results contrasted with the bone defects found after traditional rigid screw fixation Latarjet, which leads to excessive bone resorption.^33^ Therefore, our animal experimental model did not fully simulate the biomechanical environment in which coracoid healing and shaping proceed after Latarjet, but this study does indicate that suture‐button fixation Latarjet has some advantages over traditional surgery. However, our findings, which created a link between the mechanical environment and SHH and CXCL12, have important implications in exploring the mechanisms by which suture‐button fixation Latarjet promotes bone healing and shaping.

Many scholars believe that the micro‐movements of the fracture site caused by stress stimulation promote bone healing, and bone healing is the result of mature lamellar bone formation from osteoblasts, while normal bone shaping is the result of the balance between osteogenesis and osteoclast.^34^, ^35^, ^36^ We speculate that micro‐movements at the fracture site may affect the metabolism of osteoblasts and osteoclasts, and the regulation of bone formation and resorption by micro‐movements, achieved by using a surgical fixation system with appropriate stress stimulation, may be the key to achieving complete bone healing. The micro‐movements at the fracture site are the result of the synergism of fixation strength and stress stimulation. Whether the biomechanical environment constructed by the suture‐button fixation Latarjet can achieve micro‐movements between the coracoid and the glenoid still needs to be experimentally demonstrated.

In summary, this study presents novel findings pertaining to the influence of flexible fixation and load‐bearing stress environment on bone healing and remodeling. However, the efficacy of suture‐button fixation Latarjet in the treatment of recurrent shoulder joint dislocation warrants further investigation, in light of the limited clinical experimental research reported to date.

## 5 CONCLUSIONS

Flexible fixation and a load‐bearing stress environment contribute to maintaining normal levels of CXCL12 and SHH, thereby facilitating optimal bone healing and shaping. This suggests that the biomechanical environment created by the suture‐button fixation Latarjet procedure may play a pivotal role in regulating the CXCL12 and SHH levels required for coracoid healing and shaping.

## AUTHOR CONTRIBUTIONS

Xingfu Li designed and supervised the manuscript. Xingfu Li wrote the manuscript. Zhenhan Deng and Wei Lu revised the manuscript. The final manuscript was reviewed and approved by all authors.

## FUNDING INFORMATION

This work was supported by the National Natural Science Foundation of China (no.82072515), Guangdong Basic and Applied Basic Research Foundation (2023A151522 0072, 2021A1515220030), Shenzhen High‐level Hospital Construction Fund (no.4001013), Shenzhen Science and Technology Projects (no. JCYJ20220530150615035).

## CONFLICT OF INTEREST STATEMENT

The authors declare that they have no conflict of interest.

## ETHICS STATEMENT

All the animal experiments were approved by the Shenzhen second people’ s hospital, China Technology Industry Holdings (Shenzhen) Co., Ltd (IRB no: 202200112) and performed in accordance with the national guidelines for animal welfare.

## References

[1] Deng Z , Wei L , Liu C , et al. Surgical considerations for glenoid bone loss in anterior glenohumeral instability: a narrative review. Eur J Trauma Emerg Surg. 2024;50(2):395‐403. 37642655 10.1007/s00068-023-02357-y

[2] Sano H , Komatsuda T , Abe H , Ozawa H , Yokobori TA Jr . Proximal‐medial part in the coracoid graft demonstrates the most evident stress shielding following the Latarjet procedure: a simulation study using the 3‐dimensional finite element method. J Shoulder Elb Surg. 2020;29(12):2632‐2639. 10.1016/j.jse.2020.03.037 32713665

[3] Laurent W , Sara DB , Alexander VT , et al. Analysis of failures after the Bristow‐Latarjet procedure for recurrent shoulder instability. Int Orthop. 2019;43(8):1899‐1907. 30151779 10.1007/s00264-018-4105-6

[4] Ernstbrunner L , Pastor T , Waltenspül M , Gerber C , Wieser K . Salvage iliac crest bone grafting for a failed Latarjet procedure: analysis of failed and successful procedures. Am J Sports Med. 2021;49(13):3620‐3627. 34523379 10.1177/03635465211040468

[5] Boileau P , Mercier N , Old J . Arthroscopic Bankart‐Bristow‐Latarjet (2B3) procedure: how to do it and tricks to make it easier and safe. Orthop Clin North Am. 2010;41(3):381‐392. 20497813 10.1016/j.ocl.2010.03.005

[6] Jian X , Haifeng L , Wei L , et al. Modified arthroscopic Latarjet procedure: suture‐button fixation achieves excellent remodeling at 3‐year follow‐up. Am J Sports Med. 2020;48(1):39‐47. 31765231 10.1177/0363546519887959

[7] Liang D , Liu H , Liang X , et al. Effect of modified arthroscopic Latarjet on Acromiohumeral distance at 5‐year follow‐up. Orthop J Sports Med. 2021;9(12):23259671211063844. 34988238 10.1177/23259671211063844 PMC8721388

[8] Deng Z , Long Z , Wei L . LUtarjet‐limit unique coracoid osteotomy Latarjet (With video). Burns Trauma. 2022;10:tkac021. 35664892 10.1093/burnst/tkac021 PMC9155144

[9] Lu W , Liang D , Liu Y , et al. Modified suture button Latarjet procedure with Coracoacromial ligament and pectoralis minor preservation achieves good clinical outcomes at 2‐year follow‐up: case series of Latarjet technique. Art Ther. 2024;S0749‐8063(24)00343‐8. 10.1016/j.arthro.2024.04.037 38735417

[10] Xia P , Shi Y , Wang X , Li X . Advances in the application of low‐intensity pulsed ultrasound to mesenchymal stem cells. Stem Cell Res Ther. 2022;13(1):214. 35619156 10.1186/s13287-022-02887-z PMC9137131

[11] Weiyong W , Zhao Z , Wang Y , et al. Biomechanical effects of mechanical stress on cells involved in fracture healing. Orthop Surg. 2024;16(4):811‐820. 38439564 10.1111/os.14026 PMC10984830

[12] Elmansi AM , Eisa NH , Periyasamy‐Thandavan S , et al. DPP4‐ truncated CXCL12 alters CXCR4/ACKR3 signaling, osteogenic cell differentiation, migration, and senescence. ACS Pharmacol Transl Sci. 2022;6(1):22‐39. 36659961 10.1021/acsptsci.2c00040 PMC9844133

[13] Serowoky MA , Kuwahara ST , Liu S , Vakhshori V , Lieberman JR , Mariani FV . A murine model of large‐scale bone regeneration reveals a selective requirement for sonic hedgehog. NPJ Regen Med. 2022;7(1):30. 35581202 10.1038/s41536-022-00225-8 PMC9114339

[14] Jen‐Chieh L , Hsin‐Pei L , Ro‐Lin CG , et al. Kefir peptides promote osteogenic differentiation to enhance bone fracture healing in rats. Life Sci. 2022;310:121090. 36257457 10.1016/j.lfs.2022.121090

[15] Sun L , An S , Zhang Z , et al. Molecular and metabolic mechanism of low‐intensity pulsed ultrasound improving muscle atrophy in Hindlimb unloading rats. Int J Mol Sci. 2021;22(22):12112. 34829990 10.3390/ijms222212112 PMC8625684

[16] Zhu W‐Y , Yang W‐F , Wang L‐l , et al. The effect of drug holiday on preventing medication‐related osteonecrosis of the jaw in osteoporotic rat model. J Orthop Translat. 2023;39:55‐62. 36721766 10.1016/j.jot.2022.12.006 PMC9860383

[17] Kaur A , Mohan S , Rundle CH . A segmental defect adaptation of the mouse closed femur fracture model for the analysis of severely impaired bone healing. Animal Model Exp Med. 2020;3(2):130‐139. 32613172 10.1002/ame2.12114 PMC7323699

[18] Kapania EM , Reif TJ , Tsumura A , Eby JM , Callaci JJ . Alcohol‐induced Wnt signaling inhibition during bone fracture healing is normalized by intermittent parathyroid hormone treatment. Animal Model Exp Med. 2020;3(2):200‐207. 32613179 10.1002/ame2.12116 PMC7323703

[19] Savaridas T , Wallace RJ , Muir AY , Salter DM , Simpson AHRW . The development of a novel model of direct fracture healing in the rat. Bone Joint Res. 2012;1(11):289‐296. 23610660 10.1302/2046-3758.111.2000087 PMC3626205

[20] Pontzer H , Lieberman DE , Momin E , et al. Trabecular bone in the bird knee responds with high sensitivity to changes in load orientation. J Exp Biol. 2006;209(Pt1):57‐65. 16354778 10.1242/jeb.01971

[21] Turner CH . Functional determinants of bone structure: beyond Wolff's law of bone transformation. Bone. 1992;13(6):403‐409. 1476817 10.1016/8756-3282(92)90082-8

[22] Xuehua L , Han L , Nookaew I , et al. Stimulation of Piezo1 by mechanical signals promotes bone anabolism. elife. 2019;8:e49631. 31588901 10.7554/eLife.49631 PMC6779475

[23] Herrmann M , Engelke K , Ebert R , et al. Interactions between muscle and bone‐where physics meets biology. Biomol Ther. 2020;10(3):432. 10.3390/biom10030432 PMC7175139 32164381

[24] Deng Z , Gao X , Sun X , Cui Y , Amra S , Huard J . Gender differences in tibial fractures healing in normal and muscular dystrophic mice. Am J Transl Res. 2020;12(6):2640‐2651. 32655796 PMC7344076

[25] Borgiani E , Figge C , Kruck B , Willie BM , Duda GN , Checa S . Age‐related changes in the mechanical regulation of bone healing are explained by altered cellular mechanoresponse. J Bone Miner Res. 2019;34(10):1923‐1937. 31121071 10.1002/jbmr.3801

[26] Sivaraj KK , Majev P‐G , Dharmalingam B , et al. Mesenchymal stromal cell‐derived septoclasts resorb cartilage during developmental ossification and fracture healing. Nat Commun. 2022;13(1):571. 35091558 10.1038/s41467-022-28142-w PMC8799643

[27] Yuhe J , Ping Z , Xiao Z , et al. Advances in mesenchymal stem cell transplantation for the treatment of osteoporosis. Cell Prolif. 2021;54(1):e12956. 33210341 10.1111/cpr.12956 PMC7791182

[28] Sharaf‐Eldin WE , Abu‐Shahba N , Mahmoud M , et al. The modulatory effects of mesenchymal stem cells on osteoclastogenesis. Stem Cells Int. 2016;2016:1908365. 26823668 10.1155/2016/1908365 PMC4707367

[29] Sanchez‐Martin L , Estecha A , Samaniego R , et al. The chemokine CXCL12 regulates monocyte‐macrophage differentiation and RUNX3 expression. Blood. 2011;117(1):88‐97. 20930067 10.1182/blood-2009-12-258186

[30] Wang J , Liu D , Guo B , et al. Role of biphasic calcium phosphate ceramic‐mediated secretion of signaling molecules by macrophages in migration and osteoblastic differentiation of MSCs. Acta Biomater. 2017;51:447‐460. 28126596 10.1016/j.actbio.2017.01.059

[31] Takebe H , Shalehin N , Hosoya A , Shimo T , Irie K . Sonic hedgehog regulates bone fracture healing. Int J Mol Sci. 2020;21(2):677. 31968603 10.3390/ijms21020677 PMC7013927

[32] Liu P , Ji T , Wang W , et al. Effects of mechanical stress stimulation on function and expression mechanism of osteoblasts. Front Bioeng Biotechnol. 2022;10:830722. 35252138 10.3389/fbioe.2022.830722 PMC8893233

[33] Di Giacomo G , Peebles LA , Midtgaard KS , et al. Risk factors for recurrent anterior glenohumeral instability and clinical failure following primary Latarjet procedures: An analysis of 344 patients. J Bone Joint Surg Am. 2020;102(19):1665‐1671. 33027119 10.2106/JBJS.19.01235

[34] Ali M , Seung‐Hwan C . Effect of initial micro‐movement of a fracture gap fastened by composite prosthesis on bone healing. Compos Struct. 2019;226:111213.

[35] Zhang JQ , Takahashi A , Gu JY , et al. In vitro and in vivo detection of tunneling nanotubes in normal and pathological osteoclastogenesis involving osteoclast fusion. Lab Investig. 2021;101(12):1571‐1584. 34537825 10.1038/s41374-021-00656-9

[36] Shah FA , Thomsen P , Palmquist A . A review of the impact of implant biomaterials on osteocytes. J Dent Res. 2018;97(9):977‐986. 29863948 10.1177/0022034518778033 PMC6055115

